# Individual and population-level responses to ocean acidification

**DOI:** 10.1038/srep20194

**Published:** 2016-01-29

**Authors:** Ben P. Harvey, Niall J. McKeown, Samuel P. S. Rastrick, Camilla Bertolini, Andy Foggo, Helen Graham, Jason M. Hall-Spencer, Marco Milazzo, Paul W. Shaw, Daniel P. Small, Pippa J. Moore

**Affiliations:** 1 Institute of Biological, Environmental, and Rural Sciences, Aberystwyth University, Aberystwyth, SY23 3DA, UK; 2 Institute of Marine Research, P.O. Box 1870, Nordnes, 5817 Bergen, Norway; 3 Ocean and Earth Science, National Oceanography Centre Southampton, University of Southampton, Waterfront Campus, European Way, Southampton SO14 3ZH, UK; 4 School of Biological Sciences, Queen’s University Belfast, Belfast, BT9 7BL, Northern Ireland, UK; 5 Marine Biology and Ecology Research Centre, School of Marine Science & Engineering, University of Plymouth, Drake Circus, Plymouth PL4 8AA, UK; 6 Uni Research Environment, P.O. Box 7810, 5020 Bergen, Norway; 7 School of Marine Science and Technology, Ridley Building, Newcastle University, Newcastle upon Tyne, NE1 7RU, UK; 8 Dipartimento di Scienze della Terra e del Mare, CoNISMa, University of Palermo, via Archirafi 28, 90123 Palermo, Italy; 9 Biology Department, St. Francis Xavier University, Antigonish, NS, B2G 2W5, Canada; 10 Centre for Marine Ecosystems Research, Edith Cowan University, Perth, Australia, 6027

## Abstract

Ocean acidification is predicted to have detrimental effects on many marine organisms and ecological processes. Despite growing evidence for direct impacts on specific species, few studies have simultaneously considered the effects of ocean acidification on individuals (e.g. consequences for energy budgets and resource partitioning) and population level demographic processes. Here we show that ocean acidification increases energetic demands on gastropods resulting in altered energy allocation, i.e. reduced shell size but increased body mass. When scaled up to the population level, long-term exposure to ocean acidification altered population demography, with evidence of a reduction in the proportion of females in the population and genetic signatures of increased variance in reproductive success among individuals. Such increased variance enhances levels of short-term genetic drift which is predicted to inhibit adaptation. Our study indicates that even against a background of high gene flow, ocean acidification is driving individual- and population-level changes that will impact eco-evolutionary trajectories.

Ocean acidification represents a deleterious anthropogenic influence that can affect the vital functions of marine organisms^1^, with many studies reporting implications for growth, development and reproduction due to energetic trade-offs in important energy-demanding processes^2^,^3^,^4^. If organisms cannot maintain their energy budgets then their reproductive contribution to subsequent generations may be compromised, with implications for species’ abundances and distributions^5^. Predicting the fate of marine biodiversity in the face of climate change, including ocean acidification, will therefore require scaling up from impacts upon individuals’ physiology and fitness to population level demographic processes.

We investigated the effects of ocean acidification on the gastropod *Hexaplex trunculus* (L.), a widespread Mediterranean mollusc. Our study made use of CO_2_ seeps off the coast of Isola Vulcano (Sicily, Italy), which provide a natural gradient of pH and a carbonate chemistry analogue for future oceans^6^, as well as the means to investigate naturally-assembled communities. As a slow-moving and predominantly direct developing organism *H. trunculus* has low dispersal capacity (relative to the spatial scale of the seep system), resulting in the potential for population genetic structuring at a fine geographical scale. This makes it an ideal model with which to investigate individual- to population-level responses to prolonged (multigenerational), exposure to elevated *p*CO_2_.

Three sites along the natural pH gradient were used (Fig. 1; full carbonate chemistry details are presented in Supplementary Table 1). All three sites experience stable ambient temperature and salinity, with the mean surface seawater pH progressively decreasing with proximity to the CO_2_ seeps^7^. The Low pH site experienced elevated *p*CO_2_ conditions (7.65 ± 0.01 pH_T_), the Control site experienced ambient pH conditions (8.00 ± 0.01 pH_T_) over the long-term, albeit with periods of short-term (hours/days) pH variability (likely associated with wind-driven currents^7^) and the Reference site experienced long-term stable, ambient pH conditions (8.07 ± 0.01 pH_T_), unaffected by the seeps.

Environmental factors modulate metabolic activity through alterations in resource demand and/or availability. Should insufficient resources be available, then stress-induced trade-offs between energy demanding processes can occur (e.g. growth, shell maintenance and reproduction)^4^. The capacity of an organism to maintain elevated metabolic rates (relative to their tissue mass^4^) may allow them to sustain positive life-history traits^8^, and determine organism fitness in future acidified oceans^4^.

Using reciprocal transplants of *H. trunculus* between two sites (Low pH and Control site) we tested how ocean acidification modulates metabolic activity using oxygen consumption rate (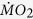) as a proxy. Results demonstrate that metabolic rate was significantly increased by exposure to elevated *p*CO_2_ (ANOVA, *F*_*1*,20_ = 4.87, *p* = 0.039). The mean 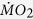 of individuals collected and caged at the Low pH site was significantly higher compared to individuals collected from, and caged in control conditions (Fig. 2). This suggests that individuals of *H. trunculus* experience increased energetic demand under persistent exposure to ocean acidification, in part this is likely due to higher predicted costs of maintaining calcification and acid–base homeostasis^9^. Both nuclear and mtDNA genetic markers showed no evidence of restricted gene flow between populations at these two sites (see Supplementary Table 2 and 3) suggesting that differences in metabolic rate were a result of acclimatisation rather than being driven by genetic differences attributable to drift or population isolation. When *H. trunculus* individuals were reciprocally transplanted between Control and Low pH sites, metabolic rates did not significantly differ (Tukey HSD *post-hoc*, *p* > 0.05), with mean 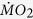 intermediate between the other two treatments (Fig. 2). These results suggest that individuals entering or leaving acidified areas may acclimate via physiological plasticity^8^ (Fig. 2).

Acclimatisation can buffer populations against the immediate impacts of ocean acidification, and even provide time for adaptation^10^. However, it can also result in stress-induced energetic trade-offs. Mean shell length (ANOVA, *F*_2,208_ = 3.33, *p* = 0.034) and thickness (ANOVA, *F*_2,66_ = 4.36, *p* = 0.017) in the acidified conditions were significantly lower (Fig. 3), but by contrast the mean (shell-free) body mass of individuals was significantly greater in the Low pH site (ANOVA, *F*_2,66_ = 22.37, *p* < 0.001; Fig. 4). Regulation of pH at the site of calcification is energetically costly^11^, and associated increases in energy partitioning towards maintaining acid–base homeostasis may reduce net calcification rates^9^. Reduced shell thickness may also lessen any growth-limiting effect that the shell structure has on somatic tissue growth^11^, allowing for a larger body mass (assuming sufficient energy is available).

When scaling from the individual to the population there is a need to consider whether responses are sex-specific, since any disproportionate effects on one or the other sex could subsequently affect population demographics. To our knowledge, no previous studies have provided evidence for skewed sex ratios associated with ocean acidification. However, it has been previously demonstrated that sex-specific effects can alter how an individual responds to, or is affected by ocean acidification. For instance, the metabolome of males and females of the blue mussel *Mytilus edulis* differed significantly when exposed to reduced pH^12^. Moreover, it has been suggested that selection pressures to ocean acidification may differ between sexes^13^. We found that significantly fewer females were present in the Low pH site (32.26%, χ^2^_1, 31_ = 3.90, *p* = 0.048), while the sex ratio in the ambient pH sites did not significantly differ from equality (Control, 54.84% female; Reference, 45.83% female, χ^2^ both *p* > 0.50). It should be noted that there are increased levels of trace elements and heavy metals at our low pH site (due to the use of the CO_2_ seep site^14^). While a previous study, at the same study sites and using the same species as the present work, found no spatial pattern in trace element bioaccumulation^15^ it is not possible to disentangle the effects of reduced pH from potential changes in sex ratios that can occur through the bioaccumulation of heavy metals and trace elements^16^,^17^. Therefore, while our findings imply that sex-specific responses to ocean acidification may occur with consequences for individual performance and fitness, as well as for population dynamics, some caution in their interpretation is advised.

Despite the absence of genetic differentiation between the sites’, the prominent feature of the genetic data was the large, significant *F*_*I*S_ values reported for the Low pH site compared to non-significant values reported for both ambient pH sites (Low pH multi-locus *F*_*IS*_ = 0.2036, *p* < 0.0001; Control multi-locus *F*_*IS*_ = −0.0368, *p* = 0.2692; Reference multi-locus *F*_*IS*_ = −0.0379, *p* = 0.9879). The *F*_*IS*_ values suggest that for *H. trunculus* some aspect of the acidified environment results in greater departures from Hardy-Weinberg equilibrium outcrossing genotype proportions in the form of heterozygote deficits. Such site-specific heterozygote deficits could be generated by inbreeding, natural selection acting on the genetic markers, or spatial/temporal structure within samples known as the Wahlund effect^18^. As the significant multi-locus *F*_*IS*_ values resulted from significant values at the majority of individual loci, locus-specific selection effects are unlikely. Inbreeding effects within a small population can also be discounted, as the Low pH site sample reported significantly reduced mean individual relatedness (mr) values compared to the Control (*p* = 0.03) and Reference (*p* = 0.048) sites (Low pH mr = −0.514; Control mr = 0.0077; Reference mr = 0.0066).

For a Wahlund effect to be observed, genetically distinct populations must occur within the geographical scale of the sampling. Due to the high gene flow indicated throughout the sampled area, any heterogeneity in allele frequencies is more likely to be generated by increased variance in reproductive success among individuals, rather than by spatial population structuring *per se*. Such variance in reproductive success among individuals has been reported for many highly fecund marine invertebrates and is often referred to as sweepstakes recruitment^19^. Within such a reproductive system many individuals do not contribute to recruitment in a given cycle, resulting in short-term genetic drift generating allele frequency differences among groups of recruits even though they are all derived from a single population. These differences among groups generate the observed heterozygote deficits and decreased mean relatedness within samples.

Variance in reproductive success among individuals may be driven by any deterministic and/or stochastic processes influencing fitness, and ocean acidification has been shown to strongly influence recruitment processes^20^. Previous studies have demonstrated that climate derived stressors may result in a reduction in the proportion of the population that are reproductively active and an increase in reproductive failure (such as in the limpet *Patella vulgata*^21^). In our study significantly reduced numbers of females in the Low pH site represents an obvious mechanism that could increase variance in reproductive success among males, however altered energy allocations and/or other cryptic factors may also contribute.

The increased variance in reproductive success and consequent genetic drift pulses (within and between cohorts) could, in theory, hinder adaptive evolution of populations in response to ocean acidification^22^,^23^. Acclimatisation can represent an important mechanism for maintaining population persistence under stressful conditions^23^. However, any energetic trade-offs may result in previously unforeseen consequences, and unless adaptive evolution can ‘catch up’^24^, there could be serious implications for population dynamics. This study demonstrates that even within a high gene flow system elevated *p*CO_2_ concentrations are driving individual and population level changes that will impact eco-evolutionary trajectories, and highlights the need for deeper understanding of the links between individual effects and (often unknown^25^) population demographics in order to predict and manage the consequences of climate change.

## Methods

### *H. trunculus* collection

Collection of *H. trunculus* (and subsequent experiments) were carried out at Levante Bay (38° 25′ N, 14° 57′ E), Vulcano, Italy in May 2013 and May 2014. During the first experimental period, individuals used for the reciprocal transplant were collected and deployed in transplantation cages, with additional individuals collected in order to measure shell length. During the second sampling period individuals were destructively sampled to determine sex, body mass (shell-free dry mass of soft tissues, including gonads) and shell thickness (outer lip). Genetic samples were collected during both experimental periods to test for temporal stability of any population structuring or demography. Samples were taken non-destructively by removing a tissue sample from the foot, then returning individuals to their respective sites.

### Reciprocal transplant experiment

Using an orthogonal design, six cages per treatment (10 × 10 × 10 cm), each containing an individual *H. trunculus*, were distributed across four treatments. Individuals from the elevated *p*CO_2_ areas were (i) transplanted into control conditions (Control site), or (ii) re-transplanted into the high *p*CO_2_ areas (Low pH site), and individuals collected from the Control site were (iii) transplanted into high *p*CO_2_ areas, or (iv) re-transplanted into the control conditions. After 14 days exposure, the cages were recovered and transported to a reservoir tank with site-specific ambient pH prior to placement into respirometers for oxygen consumption rate measurements within 30 min of collection.

### **Measurement of** 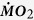


Oxygen consumption rate (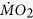) was determined using stop-flow respirometers (volume 460 ml). The respirometers were immersed in a reservoir tank (40 × 60 × 30 cm) to maintain natural temperature (19.53 ± 0.06 °C), and supplied with fully-oxygenated filtered seawater from either the Control (pH 8.1 ± 0.1) or Low pH (pH 7.8 ± 0.1) site. Individuals of *H. trunculus* were placed into a respirometer, and allowed to settle to the experimental conditions for 1 h. Water samples (taken via syringe from the respirometers) were collected in order to measure the partial pressure of oxygen (*p*O_2_), by passing the water sample over a *p*O_2_ electrode (E101/E5046 polarographic O_2_ electrode, Loligo systems, Denmark) connected to an oxygen meter (Oxygen Meter model 781, SI Strathkelvin Instruments Limited, Scotland) that was calibrated to 100% aerated site-specific seawater. The first water sample was taken following the 1 h settling period, and at the same time, the circulation of seawater from the reservoir to the respirometers was stopped. A second water sample was taken from each respirometer after 1h (maintained above 17 kPa), and the oxygen consumption rate was determined as the change in *p*O_2_ between the first and second water samples. This was multiplied by the solubility coefficient for oxygen, adjusted for salinity and temperature^26^, and the volume of water within each respirometer. Oxygen consumption rates were corrected for standard conditions of temperature and pressure (STPD), and presented as nmol O_2_ h^−1^ mg^−1^ (wet weight) STPD.

### Shell length, sex determination and body mass

Shell length was measured using a digital calliper (±0.1 mm) measuring from the apex to the tip of the siphonal canal (*n* = 73–85 per site). A macroscopic maturation scale was employed to determine the sex of *H. trunculus*^27^ (*n* = 24–31 per site). Body mass was determined by dissecting the body tissue from the shell (*n* = 24–31 per site), and measured as dry weight (g, 60 °C, 48 hours). Shell thickness (outer lip) was measured in the growing tip along the anterior portion of the shell (*n* = 18 per site), using a dissection microscope with 10× eyepiece graticule (M5A, Wild Heerbruug, Switzerland).

### Statistical analyses

Statistical analyses were performed using R. For shell lengths and thickness, differences were tested by one-way analysis of variance (ANOVA) by Site. For 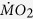, differences were tested by two-way ANOVA by Origin х Exposure, and for body mass, two-way ANOVA by Site × Sex. All significant ANOVA results were followed by Tukey’s *post-hoc* tests. Deviation from equal sex ratios were determined using Pearson’s chi-squared tests. All data sets were normally distributed and had equal variances.

### DNA extraction and genotyping

DNA was extracted from ethanol preserved tissue samples using a standard CTAB-chloroform/isoamylalcohol method. Novel microsatellite markers specific to *H. trunculus* were developed by construction of a microsatellite-enriched genomic library following methods outlined in refs^28^,^29^.

Individuals collected at two times from each of the three sites (temporal sample 1: *n* = 48 per site; temporal sample 2: *n* = 28–30 per site) were genotyped at four microsatellite loci (GenBank KJ765703-KJ765706) by electrophoresis of PCR products on an ABI3500 automated sequencer (Applied Biosystems, Foster City, California, USA), with allele sizes determined using GENEMAPPER^30^. Mitochondrial DNA (mtDNA) was assessed by PCR amplification and sequencing of a 602bp fragment of the cytochrome oxidase I gene, which was amplified using species-specific primers HtrucF (5′-ATATGGTCAGGGCTTGTTGG-3′) and HtrucR (5′-CCTGCAGGATCAAAGAACG-3′) designed from GenBank sequences (EU391577). Sequencing was performed with the PCR primers using BigDye technology (Applied Biosystems). Sequences were edited, aligned and trimmed to a standard length (532bp) in BIOEDIT^31^.

### Population genetics statistics

Deviations from Hardy-Weinberg equilibrium were evaluated using exact tests in GENEPOP^32^ and an estimator of standardised genetic variance within populations, *F*_IS_^33^, calculated in FSTAT^34^. Genetic differences among samples were quantified by *F*_ST_ (Φ) using FSTAT. FSTAT was used to test for significant differences (1000 permutations) in genetic variability (as described by *Ar*, *H*_*S*_, *H*_*E*_, *F*_IS_, *F*_ST_, relatedness, corrected relatedness) between groups.

MtDNA variation was assessed in ARLEQUIN^35^ using indices of haplotype and nucleotide diversity (*h* and *π*, respectively) and their variances, with differentiation among samples tested by global and pairwise *Φ*_*ST*_.

## Additional Information

**How to cite this article**: Harvey, B. P. *et al.* Individual and population-level responses to ocean acidification. *Sci. Rep.* **6**, 20194; doi: 10.1038/srep20194 (2016).

## Supplementary Material

Supplementary Information

## Figures and Tables

**Figure 1:**
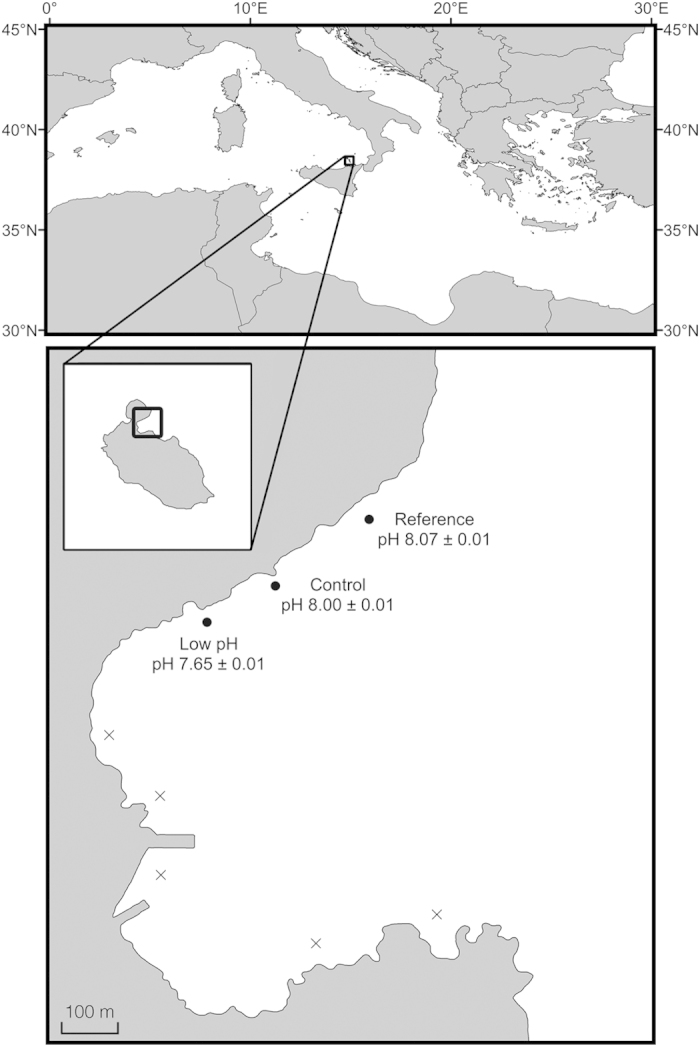
Map of the study area. Baia di Levante (Isola Vulcano, Sicily), showing sampling sites ‘Low pH’ (pH_T_ 7.65 ± 0.01), ‘Control’ (pH_T_ 8.00 ± 0.01) and ‘Reference’ (pH_T_ 8.07 ± 0.01), with ‘x’ indicating the gas seeps. The map was generated using ArcGIS 9.3 software (http://www.esri.com/software/arcgis/).

**Figure 2:**
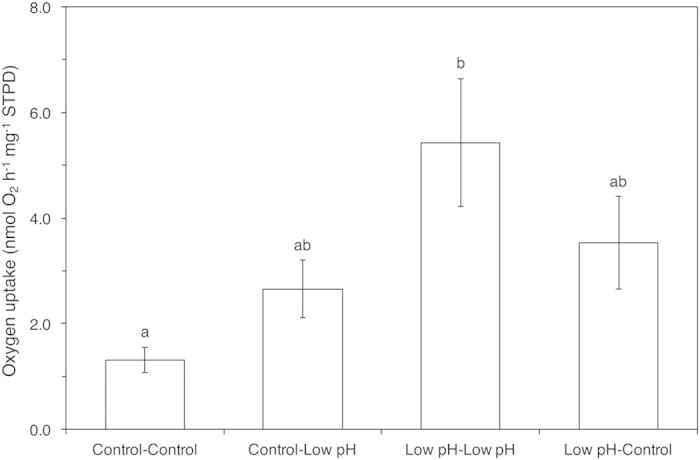
Effect of exposure to different *p*CO_2_/pH conditions on the mean (± S.E.) oxygen consumption rate of *H. trunculus* that were either (i) collected in the Control site and re-transplanted in the Control site (Control-Control), (**ii**) transplanted from the Control site to the Low pH site (Control-Low pH), (**iii**) re-transplanted within Low pH site (Low pH-Low pH) and (**iv**) transplanted from Low pH into the Control Site (Low pH-Control). Mean 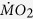 is expressed as nmol O_2_ h^−1^ mg^−1^ (WW) STPD. Significantly different treatments are indicated by different lower case letters above the column (Tukey HSD, p ≤ 0.05).

**Figure 3:**
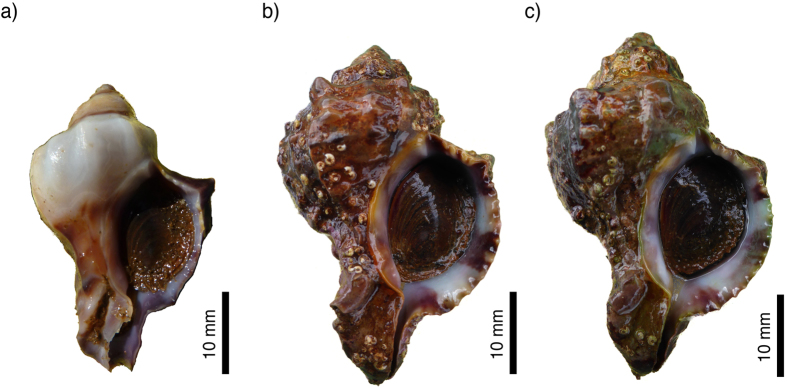
Shells of the investigated species. Samples of *H. trunculus* living at the Low pH site (**a**) showing shell dissolution and reduced shell size when compared to individuals from the Control (**b**), and Reference site (**c**). The individuals displayed are representative for both the mean shell length, as well as the overall shell condition and shape based on geometric morphometric analyses (Harvey, unpublished data).

**Figure 4:**
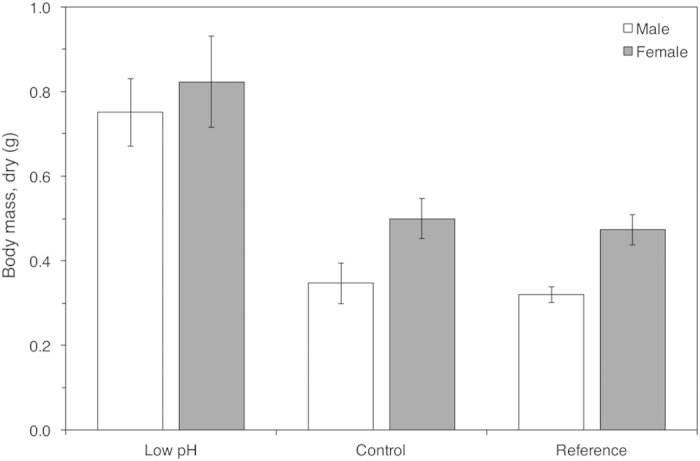
Effect of exposure to different *p*CO_2_/pH conditions on the mean (±S.E.) dry shell-free body mass of male (open bars) and female (grey bars) individuals (length 44 ± 1.2 mm [S.E.]) from the Low pH, Control and Reference site.
