# Supplementary material for: Individual and population-level responses to ocean acidification
Source: Sci Rep. 2016 Jan 29;6:20194. doi: 10.1038/srep20194 (PMC4731747; doi:10.1038/srep20194)
Supplement: Supplementary Information [file srep20194-s1.pdf]

Individual and population-level responses to ocean acidification

Ben P. Harvey, Niall J. McKeown, Samuel P.S. Rastrick, Camilla Bertolini, Andy Foggo, Helen Graham, Jason M. Hall-Spencer, Marco Milazzo, Paul W. Shaw, Daniel P. Small, Pippa J. Moore

Supplementary Information

Measurements of seawater carbonate chemistry. Seawater pH, temperature, and salinity were measured at the three sites (Low pH, Control and Reference) on numerous occasions throughout the observations and experiments described in the main methods section. During the reciprocal transplants, measurements were taken twice daily in both the Low pH and Control sites, with measurements taken in the Reference site every 1-3 days. Total alkalinity (TA) was measured once weekly from all three sites. Measurements for all carbonate chemistry monitoring were taken as follows: pH_{NBS} (Seven Easy pH InLab micro-electrode coupled to a Sevengo pH meter, Mettler-Toledo Ltd., Beaumont Leys, UK), temperature (digital thermometer, HH806AU, OMEGA Eng. Ltd., Manchester, UK), salinity (hand-held conductivity meter, TA 197 LFMulti350, WTW, Weilheim, Germany) and A_T (Hanna HI 755 Alkalinity Checker, Leighton Buzzard, UK).

In order to calculate the additional carbonate chemistry parameters, following Nisumaa et al. (ref [1]), the dissolved inorganic carbon (DIC) was calculated using the software CO2SYS[2], with the measured pH_{NBS} and A_T as the input variables. Subsequently, the additional carbonate system parameters were calculated using the R package seacarb[3] using the calculated C_T and measured A_T. For both CO2SYS and seacarb, we used disassociation constants from Mehrbach et al. (ref [4]), as adjusted by Dickson and Millero (ref [5]), and KSO_4 using Dickson (ref [6]) (Table S1).

Table S1. Seawater properties (Mean ± S.E.) at the three sites (Low pH, Control, Reference). pH_T, temperature, salinity, and total alkalinity (A_T) are measured values. Seawater pCO_2, dissolved inorganic carbon (DIC), bicarbonate (HCO_3^-), carbonate (CO_3^{2-}), carbon dioxide (CO_2), saturation states for calcite ($\Omega_{calcite}$) and aragonite ($\Omega_{aragonite}$) are values calculated using the carbonate chemistry system analysis program CO2SYS[2] and the R package seacarb[3].

	Low pH	Control	Reference
**pH_T**	7.65 ± 0.003	8.00 ± 0.001	8.07 ± 0.003
**Temp (℃)**	19.527 ± 0.01	19.527 ± 0.01	19.542 ± 0.004
**Salinity (psu)**	38.186 ± 0.01	38.189 ± 0.01	38.141 ± 0.01
**TA (μmol kg^{-1})**	2557.05 ± 0.12	2623.16 ± 0.19	2533.64 ± 0.97
**pCO_2 (μatm)**	1535.57 ± 18.80	531.73 ± 2.70	407.33 ± 4.05
**DIC (μmol kg^{-1})**	2450.26 ± 1.24	2341.98 ± 0.71	2221.98 ± 2.16
**HCO_3^- (μmol kg^{-1})**	2293.60 ± 1.20	2119.27 ± 1.07	1987.65 ± 3.27
**CO_3^{2-} (μmol kg^{-1})**	107.09 ± 0.48	205.58 ± 0.45	221.23 ± 1.33
**CO_2 (μmol kg^{-1})**	49.57 ± 0.62	17.13 ± 0.09	13.10 ± 0.13
$\Omega_{calcite}$	2.50 ± 0.01	4.80 ± 0.01	5.16 ± 0.03
$\Omega_{aragonite}$	1.63 ± 0.01	3.12 ± 0.01	3.36 ± 0.02

Table S2. Descriptive statistics for the samples analysed by microsatellite (Locus A-D) and mtDNA (COI) for the three sites (Low pH, Control, Reference) for the first temporal sample (1), the second temporal sample (2) and the two temporal samples together (Pooled). For microsatellites, observed (H_O) and expected (H_E) heterozygosity, allele numbers (N_A) and p-values for tests of fit to Hardy-Weinberg equilibrium genotype expected proportions (*p*HW). *p*-values in bold denotes values $<$ 0.05. For mtDNA, haplotype (h) and nucleotide (π) diversities and associated standard deviations (SD).

		Low pH			Control			Reference		
		1	2	Pooled	1	2	Pooled	1	2	Pooled
**Microsatellite**										
**Locus A**	H_O	0.55	0.571	0.559	0.765	0.552	0.667	0.683	0.7	0.69
	H_E	0.716	0.685	0.702	0.675	0.612	0.644	0.672	0.657	0.664
	N_A	4	4	4	4	4	4	4	4	4
	pHW	0.111	0.282	0.105	0.453	0.118	0.733	0.941	1	0.788
	F_{IS}	0.234	0.168	0.205	-0.136	0.1	-0.035	-0.018	-0.067	-0.04
**Locus B**	H_O	0.45	0.5	0.471	0.735	0.586	0.667	0.561	0.567	0.563
	H_E	0.592	0.642	0.613	0.574	0.528	0.552	0.545	0.579	0.557
	N_A	3	3	3	3	4	4	3	3	3
	pHW	**0.038**	**0.007**	**0.001**	0.129	0.898	0.156	**0.036**	0.634	0.64
	F_{IS}	0.242	0.225	0.233	-0.287	-0.114	-0.209	-0.029	0.022	-0.012
**Locus C**	H_O	0.5	0.393	0.456	0.758	0.696	0.732	0.78	0.769	0.776
	H_E	0.672	0.669	0.671	0.716	0.7	0.718	0.797	0.749	0.782
	N_A	6	6	6	6	7	7	6	6	6
	pHW	**0.027**	**0.001**	**0.001**	0.341	0.436	0.939	0.581	0.734	0.916
	F_{IS}	0.259	0.418	0.322	-0.059	0.007	-0.021	0.021	-0.028	0.008
**Locus D**	H_O	0.711	0.643	0.682	0.677	0.69	0.683	0.771	0.862	0.813
	H_E	0.741	0.712	0.727	0.765	0.725	0.743	0.729	0.768	0.761
	N_A	6	6	7	6	7	7	7	8	8
	pHW	0.265	0.112	0.058	0.069	0.487	0.068	0.831	0.723	0.911
	F_{IS}	0.042	0.098	0.063	0.116	0.049	0.081	-0.059	-0.125	-0.068
**mtDNA**										
**COI**	***h (SD)***	0.5115 (0.0777)	0.3836 (0.1133)	0.4636 (0.068)	0.5101 (0.0749)	0.3624 (0.0995)	0.4528 (0.061)	0.274 (0.0855)	0.3425 (0.0971)	0.3001 (0.064)
	**π (SD)**	0.0095 (0.0052)	0.004 (0.0026)	0.0074 (0.0042)	0.0084 (0.0047)	0.0075 (0.0043)	0.008 (0.0044)	0.0058 (0.0034)	0.0072 (0.0042)	0.0063 (0.0036)

Table S3. Pairwise genetic differentiation (across loci) between sites (Low pH, Control and Reference) for a) temporal sample 1, b) temporal sample 2, and c) temporal samples 1 and 2 pooled. The lower triangular matrix of each square reports the unbiased F_{ST} estimator[7], and the upper triangular matrix of each square reports p-value of the respective exact test of allele frequency homogeneity.

a) Temporal sample 1

	**Low pH**	**Control**	**Reference**
**Low pH**		$p = 0.88$	$p = 0.033$
**Control**	-0.0026		$p = 0.033$
**Reference**	0.0194	0.0165	

b) Temporal sample 2

	**Low pH**	**Control**	**Reference**
**Low pH**		$p = 0.28$	$p = 0.43$
**Control**	0.0086		$p = 0.33$
**Reference**	0.0023	0	

c) Samples pooled

	**Low pH**	**Control**	**Reference**
**Low pH**		$p = 0.08$	$p = 0.067$
**Control**	0.0069		$p = 0.067$
**Reference**	0.0093	0.0074	

Mitochondrial Markers. Pairwise tests of Φ_{ST} demonstrated no significant differences between the sites (Low pH:Control = -0.0126, p = 0.80, Low pH:Reference = -0.0047, p = 0.39, Control:Reference = -0.0100, p= 0.58) Therefore, the mtDNA homogeneity among sites is concordant with the nuclear (microsatellite) pattern, supporting the observation that there is no significant breakdown in gene flow between sites.

Table S4. Tukey HSD *post-hoc* test results (following the analysis of variance) for the effect of exposure to different pCO$_2$/pH conditions on the mean (± S.E.) oxygen consumption rate of *H. trunculus*. Pairwise differences in mean $\dot{M}O_2$ (expressed as nmol O_2 h^{-1} mg^{-1} (WW) STPD) and associated p-values between individuals that were either (i) collected in the Control site and re-transplanted in the Control site (Control-Control), (ii) transplanted from the Control site to the Low pH site (Control-Low pH), (iii) re-transplanted within Low pH site (Low pH-Low pH) and (iv) transplanted from Low pH into the Control Site (Low pH-Control).

**Comparison 1**	**Comparison 2**	**Difference**	***p*-value**
Control:Control	Control:Low pH	-6.73	0.576
Control:Control	Low pH:Control	-11.10	0.175
Control:Control	Low pH:Low pH	-20.58	0.004
Control:Low pH	Low pH:Low pH	-13.84	0.066
Low pH:Control	Control:Low pH	4.37	0.834
Low pH:Control	Low pH:Low pH	-9.47	0.292

References

1. Nisumaa, A.-M. *et al.* EPOCA/EUR-OCEANS data compilation on the biological and biogeochemical responses to ocean acidification. *Earth. Sys. Sci. Data* **2**, 167–175 (2010).

2. Lewis, E. & Wallace, D.W.R. *Program developed for CO_2 system calculations, ORNL/CDIAC-105*. (Carbon Dioxide Information Analysis Center, Oak Ridge National Laboratory, U.S. Department of Energy, Oak Ridge, Tennessee, U.S., 1998).

3. Lavigne, H. & Gattuso, J-P. *Seacarb: seawater carbonate chemistry with R, R package version 3. http://cran.r-project.org/web/packages/seacarb/*. 2010).

4. Mehrbach, C., Culberson, C.H., Hawley, J.E. & Pytkowicz, R.M. Measurement of the apparent dissociation constants of carbonic acid in seawater at atmospheric pressure. *Limnol. Oceanogr.* **18**, 897–907 (1973).

5. Dickson, A.G. & Millero, F.J. A comparison of the equilibrium constants for the dissociation of carbonic acid in seawater media. *Deep-Sea Res. Part I Oceanogr. Res. Pap.* **34**, 1733–1743 (1987).

6. Dickson, A.G. Thermodynamics of the dissociation of boric acid in potassium chloride solutions from 273.15 to 318.15 K.. *J. Chem. Eng. Data* **35**, 253–257 (1990).

7. Weir, B. S. & Cockerham, C. C. Estimating F-statistics for the analysis of population structure. *Evolution* 1358–1370 (1984).
